# Tumor necrosis factor-like weak inducer of apoptosis induces inflammation in Graves’ orbital fibroblasts

**DOI:** 10.1371/journal.pone.0209583

**Published:** 2018-12-21

**Authors:** Sung Jun Lee, Jinjoo Kim, JaeSang Ko, Eun Jig Lee, Hyoung Jun Koh, Jin Sook Yoon

**Affiliations:** 1 Yonsei Bon Eye Clinic, Seoul, Korea; 2 Department of Ophthalmology, Institute of Vision Research, Severance Hospital, Yonsei University College of Medicine, Seoul, Korea; 3 Department of Endocrinology, Severance Hospital, Yonsei University College of Medicine, Seoul, Korea; Tsinghua University School of Life Sciences, CHINA

## Abstract

Tumor necrosis factor-like weak inducer of apoptosis (TWEAK), along with its receptor fibroblast growth factor-inducible (Fn)14, is associated with various biological activities including inflammation. However, its role in the pathogenesis of Graves’ orbitopathy (GO) is unknown. In this study, we investigated the mechanism by which TWEAK regulates inflammatory signaling in orbital fibroblasts from GO patients. We found that *TWEAK* and *tumor necrosis factor-α* (*TNFA*) mRNA levels were upregulated in GO as compared to non-GO tissue samples. *TWEAK*, *TNF receptor* (*TNFR*)*1*, *TNFR2*, and *TNFR superfamily member 12A* mRNA, and TWEAK and Fn14 protein levels were increased by interleukin (IL)-1β and TNF-α treatment. Treatment with exogenous recombinant TWEAK increased the transcript and protein expression of the pro-inflammatory cytokines IL-6, IL-8, and monocyte chemoattractant protein-1 to a greater extent in GO than in non-GO cells, while treatment with the anti-Fn14 antibody ITEM4 suppressed TWEAK-induced pro-inflammatory cytokine release and hyaluronan production. Additionally, the serum level of TWEAK was higher in Graves’ disease patients with (341.86 ± 86.3 pg/ml) as compared to those without (294.09 ± 41.44 pg/ml) GO and healthy subjects (255.33 ± 39.38 pg/ml), and was positively correlated with clinical activity score (r = 0.629, P < 0.001) and thyroid binding immunoglobulin level (r = 0.659, P < 0.001). These results demonstrate that TWEAK/Fn14 signaling contributes to GO pathogenesis. Moreover, serum TWEAK level is a potential diagnostic biomarker for inflammatory GO, and modulating TWEAK activity may be an effective therapeutic strategy for suppressing inflammation and tissue remodeling in GO.

## Introduction

Graves’ orbitopathy (GO) is an inflammatory autoimmune disorder of the orbit associated with Graves’ disease (GD) that occurs concurrently with thyroid dysfunction in about 40% of cases[1]. An epidemiological study in the United States reported a GO incidence of 16/100,000 in femaile and 2.9/100,000 in male [2], and among 237 GD patients seen at a single center in Europe, 81.9% had no GO at baseline but 2.6% progressed to moderate-to severe GO, and among 237 GD patients seen at a single center in Europe, 81.9% had no GO at baseline but 2.6% progressed to moderate-to-severe GO. Thyroid-stimulating hormone receptor (TSHR) expressed in orbital fibroblasts is an autoimmune target in GO [3–6]. The binding of auto-antibodies to orbital fibroblasts activates T cell-dependent inflammatory processes; activated cluster of differentiation (CD)4^+^ T cells secrete interleukin (IL)-1, interferon-γ, and tumor necrosis factor (TNF)-α, inducing the expression of TSHR and CD40 on the surface of orbital fibroblasts and promoting the secretion of IL-6, IL-8, fibronectin, type I collagen, and glycosaminoglycans [7–9]. Interaction with CD4^+^ T cells enhances Graves’ orbital fibroblast activation, proliferation, differentiation, and fat accumulation; expansion of the fibroblast pool is characterized not only by marked infiltration of activated lymphocytes, but also by increased production of hydrophilic hyaluronan in response to cytokines [10,11].

Tumor necrosis factor-like weak inducer of apoptosis (TWEAK) is an apoptosis-inducing ligand belonging to TNF superfamily that modulates various biological processes via its receptor fibroblast growth factor-inducible (Fn)14, which is encoded by *TNF receptor superfamily member* (*TNFRSF*)*12A*. TWEAK regulates inflammation, cell proliferation, angiogenesis, and tissue repair and regeneration [12–14] and induces the production of pro-inflammatory molecules—e.g., matrix metalloproteinase (MMP)1, IL-6, IL-8, monocyte chemotactic protein (MCP)-1, and regulated upon activation normal T cell—that are expressed and secreted by synoviocytes and fibroblasts as well as intercellular adhesion molecule (ICAM)-1, E-selectin, IL-8, and MCP-1 by endothelial cells [14–17]. The pro-inflammatory effects of TWEAK/Fn14 are mediated by several signaling pathways including nuclear factor (NF)-κB, mitogen-activated protein kinase (MAPK), extracellular signal-related kinase (ERK)1/2, c-Jun N-terminal kinase (JNK)1/2, and p38 cascades [18]. The TWEAK/Fn14 interaction is particularly important in synovial inflammation associated with rheumatoid arthritis (RA); higher serum levels of TWEAK, TNF-α, and IL-6 have been reported in RA patients as compared to normal controls [19]. Furthermore, recombinant (r)TWEAK enhanced the production of MCP-1 and macrophage inflammatory protein-2 by synovial cells in collagen-induced arthritis (CIA) mice, whereas a monoclonal antibody (mAb) against TWEAK attenuated synovial proliferation and inflammatory cell accumulation in CIA [20]. Thus, blocking TWEAK signaling suppresses inflammation; in fact, studies of TWEAK and Fn14 knockout mice have shown that this could be an effective treatment strategy for autoimmune diseases such as GO.

The present study investigated the mechanism by which TWEAK/Fn14 signaling contributes to the pathogenesis of inflammation in primary cultures of orbital fibroblasts from GO patients. We found that pro-inflammatory cytokine and hyaluronan production by GO fibroblasts in primary cultures was increased in response to exogenous rTWEAK, an effect that was abrogated by anti-Fn14 mAb. Moreover, serum TWEAK concentration was correlated with clinical activity score (CAS) and TSHR Ab level in GO patients. Our data suggest that TWEAK/Fn14 signaling plays a critical role in the pathogenesis of GO-related inflammation.

## Materials and methods

### Reagents and chemicals

Recombinant TWEAK, recombinant human IL-1β, and TNF-α were purchased from R&D Systems (Minneapolis, MN, USA). Dulbecco’s Modified Eagle’s Medium (DMEM), fetal bovine serum (FBS), penicillin, and gentamycin were from Hyclone Laboratories (Logan, UT, USA). Anti-Fn14 mAb (ITEM4) was from eBioscience (SanDiego, CA, USA). Inhibitors of MAPK kinase 1 (PD98059), p38 MAPK (SB203580), JNK (SP600125), phosphoinositide 3-kinase (PI3K; LY294002), and NF-κB p65 (SC514) were purchased from Calbiochem (La Jolla, CA, USA).

### Subjects and tissue and cell preparation

Orbital tissue specimens were collected from patients during orbital decompression surgery for severe GO (n = 11; eight women and three men, aged 39–55 years). Normal orbital tissue was obtained over the course of orbital surgery for other non-inflammatory diseases from age- and sex-matched control subjects with no history of autoimmune thyroid disease or GO (n = 7; four women and three men, aged 34–53 years). The non-GO subjects underwent orbital wall fracture surgery (n = 3), evisceration (n = 2), and orbital benign mass excision (n = 2). The euthyroid status of GO patients was determined at the time of surgery, and patients had not received steroid or radiation therapy for at least 3 months. Clinical information on GO and non-GO patients is shown in Table 1.

**Table 1:** Clinical information of patient samples used in an in vitro study.

Age (years)	sex	CAS	Smoker	Duration of GO (years)	Proptosis R/L (mm)	Surgery performed
GO patients						
46	M	3/7	y	1.6	20/21	Decompression
39	F	2/7	y	2.0	22/22	Decompression
40	M	0/7	n	2.2	21/20.5	Decompression
42	F	1/7	n	3.0	23/23	Decompression
48	F	0/7	n	3.2	18/18	Decompression
51	F	1/7	n	2.6	20/20	Decompression
52	M	2/7	y	1.5	24/24	Decompression
48	F	4/7	y	2.1	22.5/21	Decompression
55	F	3/7	n	1.8	22/22	Decompression
44	F	1/7	n	2.1	20.5/21	Decompression
42	F	0/7	y	2.4	23/23.5	Decompression
Non-GO control subjects						
43	M	0	y	n/a	n/a	evisceration
44	F	0	y	n/a	n/a	orbital wall fracture
34	F	0	n	n/a	n/a	evisceration
53	M	0	n	n/a	n/a	orbital wall fracture
38	F	0	n	n/a	n/a	orbital wall fracture
47	M	0	n	n/a	n/a	orbital mass excision
42	F	0	n	n/a	n/a	orbital mass excision

CAS, clinical activity score; GO, Graves’ orbitopathy; n/a, not applicable.

Primary cultures of orbital fibroblasts were established as previously described [21]. Briefly, orbital tissue explants were minced and placed in plastic culture dishes in DMEM:F12 medium with 20% FBS and antibiotics. After orbital adipose tissue had grown out from the explants, monolayers were serially passaged following trypsin/EDTA treatment and cells were incubated in DMEM with 10% FBS and antibiotics. Cells between the second and fifth passages were used for experiments.

Sera for analysis of TWEAK levels were obtained from 56 GO patients, 35 GD patients without GO, and 39 healthy control subjects (S1 Table). All patients were recruited from the Department of Ophthalmology at Severance Hospital, Seoul, Korea, and healthy controls were age and sex-matched healthy control subjects. The institutional review board of Severance Hospital, Yonsei University College of Medicine (Seoul, Korea) approved the study and written, informed consent was obtained from all participants after they had been provided with an explanation of the nature and possible consequences of the study. This study protocol adhered to the tenets of the Declaration of Helsinki.

CAS is based on the classical signs of inflammation and comprises seven items that are scored from 0 to 7. TSHR Ab level was measured using third-generation thyrotropin-binding inhibitory immunoglobulin (TBII) with the automated Cobas electrochemiluminescence immunoassay (Elecsys; Roche Diagnostics GmbH, Penzberg, Germany) according to the manufacturer’s instructions.

### Real-time polymerase chain reaction (PCR)

Cells were cultured in 6-cm dishes until they reached confluence, at which time the culture medium was replaced with serum-free DMEM. The cells were incubated for 24 h with either 10 μg/ml TNF-α or 10 μg/ml IL-1β. Total RNA was isolated and 1 μg of RNA was reverse transcribed into complementary (c)DNA according to the manufacturer’s instructions. The cDNA was used as the template for PCR amplification on an ABI StepOnePlus real-time PCR thermocycler (Applied Biosystems, Foster City, CA, USA) using SYBR Green PCR reagent (Applied Biosystems) and primers specific for *TWEAK*, *TNFRSF12A*, *TNFA*, *TNF receptor* (*TNFR)1*, *TNFR2*, *glyceraldehyde 3-phosphate dehydrogenase* (*GAPDH*).

In separate experiments, PCR was performed using TaqMan universal PCR master mix to quantitatively assess the transcript levels of *IL6*, *IL8*, *MCP1*, *ICAM1*, *MMP1*, *MMP2*, *MMP7*, *MMP9*, and *GAPDH* in cell samples using the primers shown in S2 Table. PCR reactions were performed in triplicate; target gene expression levels were normalized to that of *GAPDH*, and the results are expressed as the fold change in cycle threshold (Ct) value relative to the control group as determined with the 2^−ΔΔCt^ method [22]. Data were included in the analysis only if Ct was < 35. Results from at least three GO samples were compared with mean values of three normal control samples.

### Western blotting

Western blot analysis was performed as previously described [21] using antibodies against TWEAK, Fn14, ERK, JNK, PI3K, v-Akt murine thymoma viral oncogene (Akt), and NF-κB (Cell Signaling Technology, Danvers, MA, USA). TWEAK and Fn14 expression was measured in orbital fibroblasts stimulated with IL-1β and TNF-α (10 ng/ml) for various times. To evaluate the role of TWEAK in inflammatory signaling, orbital fibroblasts were treated with TWEAK for different times and the relative amount of each protein was quantified by densitometric analysis of immunoreactive bands using ImageJ software (National Institutes of Health, Bethesda, MD, USA), with values normalized to that of the β-actin signal of the same sample.

### Enzyme-linked immunosorbent assay (ELISA)

Proinflammatory cytokine, hyaluronan, and TWEAK levels in the culture supernatant of confluent orbital fibroblasts or in serum were determined using commercially available ELISA kits (IL-6, IL-8, MCP-1, and hyaluronan: R&D Systems; TWEAK: Bender Medsystems, Vienna, Austria) according to the manufacturers’ instructions. To investigate whether TWEAK-induced cytokine production is dependent on Fn14, cells were incubated with the Fn14-specific mAb ITEM4 (2.5 μg/ml) prior to stimulation. For comparison, GO cells were exposed to the signaling pathway inhibitors PD98059, SP600125, SB203580, and LY294002 (20 μM) and SC514 (10 μM) for 1 h prior to stimulation with TWEAK. The production of hyaluronan induced by TWEAK was analyzed in the same manner. Hyaluronan concentration in the sample was determined from a standard binding curve generated with known amounts of hyaluronan. Samples were diluted 1:10 before analysis, and the mean value of triplicate samples is reported.

To analyze serum level of TWEAK, blood samples were drawn into test tubes containing 10% (v/v) sodium citrate. Platelet-free plasma was obtained by centrifugation at 3000 × *g* for 15 min at room temperature and stored at −80°C until analysis. Serum level of TWEAK protein (pg/ml) was measured using a commercial ELISA kit. Each sample was tested three times. All serum samples were tested in the same assay.

### Statistical analysis

Experiments were performed using cell samples from different donors, which were assayed in duplicate. The mean and standard deviation were calculated from normalized measurements of each mRNA or protein from multiple (at least three) samples harvested from different individuals. Data from a single cell group at different drug concentrations and incubation times were evaluated with the Student t test or by analysis of variance using SPSS v.20.0 software (SPSS Inc., Chicago, IL, USA). Pearson’s correlation coefficient was used to analyze the relationship between serum TWEAK concentration, CAS, and TBII level. A P value < 0.05 was considered statistically significant.

## Results

### Expression of TWEAK and its receptor in orbital tissue and fibroblasts

**Fig 1:**
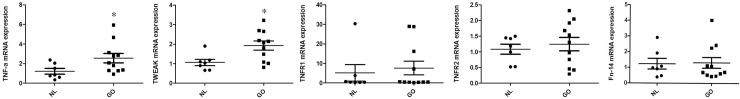
*TNFA*, *TWEAK*, *TNFR1*, *TNFR2*, and *TNFRSF12A* (Fn14) expression in GO and non-GO orbital tissues. Transcript levels of *TNFA* as well as *TWEAK* and its receptors *TNFR1*, *TNFR2*, and *TNFRSF12A* (Fn14) were compared between GO (n = 11) and non-GO (n = 7) orbital tissues by real-time PCR. *TNFA* and *TWEAK* were upregulated in GO as compared to non-GO tissue. *P < 0.05 vs. non-GO control. NL, normal non-GO subjects.

To evaluate the expression of TWEAK and its receptor in GO, orbital tissue explants were obtained from GO patients (n = 11) and non-GO healthy controls (n = 7), and relative *TNFA*, *TWEAK*, *TNFR1*, *TNFR2*, and *TNFRSF12A* mRNA levels were analyzed by real-time PCR. *TWEAK* and *TNFA* transcripts were upregulated in GO as compared to non-GO control tissue (P < 0.05; Fig 1). There were no differences in the levels of other receptors or of *TNFRSF12A* between the two groups, and *TNFR1* was expressed at a similarly low level in all samples.

**Fig 2:**
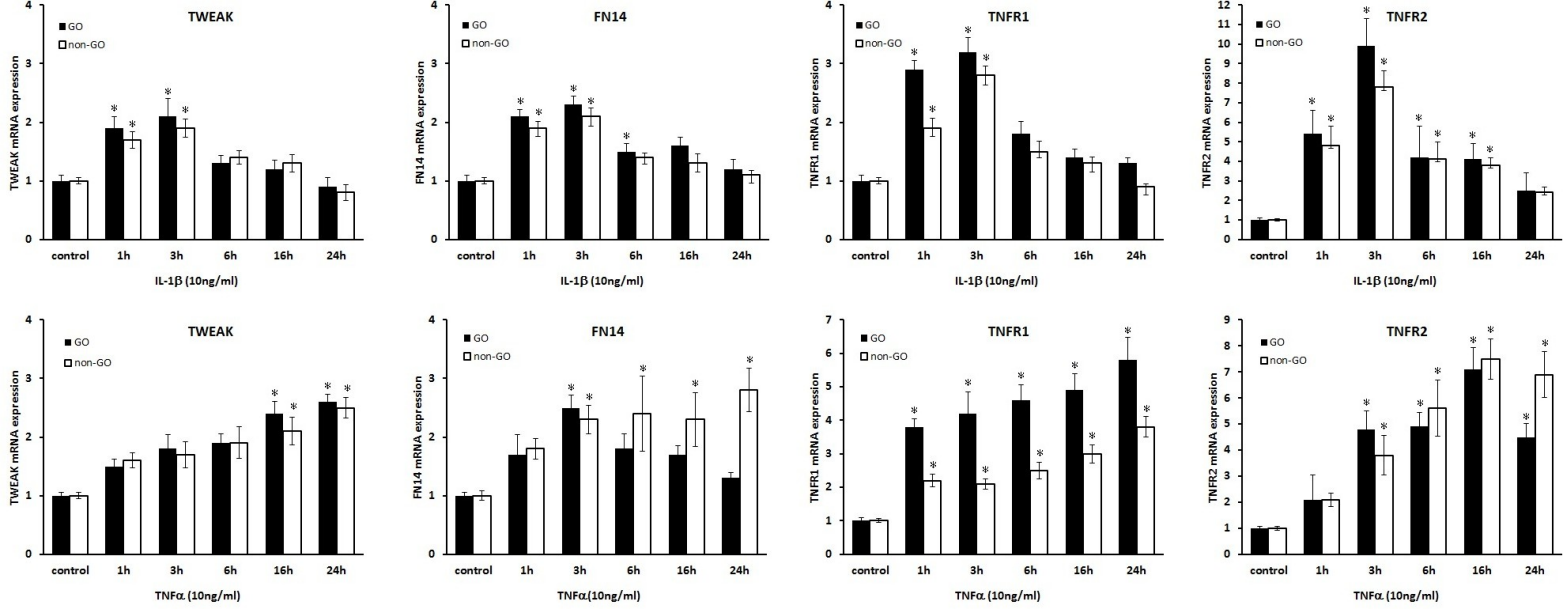
Effect of IL-1β and TNF-α on TWEAK and Fn14 expression in GO and non-GO orbital fibroblasts. Cultured GO (n = 3, black columns) and non-GO (n = 3, white columns) orbital fibroblasts were treated with IL-1β (10 ng/ml, first row) or TNF-α (10 ng/ml, second row) for 1, 3, 6, 16, or 24 h. Data in the columns represent the mean fold difference in relative mRNA level ± standard deviation of triplicate measurements. *P < 0.05 vs. untreated cells.

**Fig 3:**
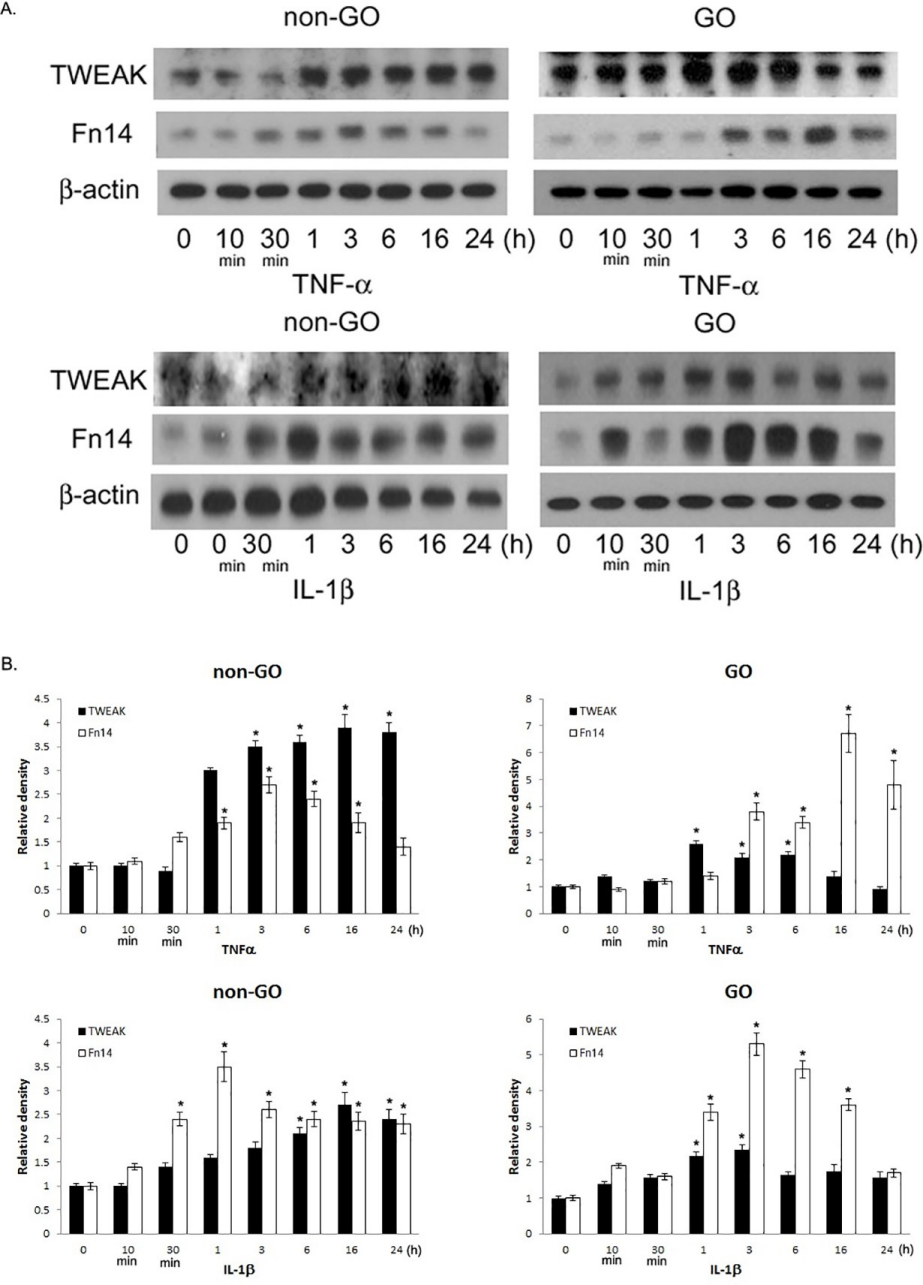
Effect of IL-1β and TNF-α on TWEAK and Fn14 protein levels in GO and non-GO orbital fibroblasts. Cultured GO (n = 3) and non-GO (n = 3) orbital fibroblasts were treated with IL-1β (10 ng/ml) or TNF-α (10 ng/ml) from 10 min to 24 h. TWEAK and Fn14 protein levels were evaluated by western blotting. (A) Representative gel images. (B) Relative amount of each protein quantified by densitometric analysis of immunoreactive bands using ImageJ software and normalized to the level of β-actin in the same sample (B). Data in the columns represent the mean relative density ratio ± standard deviation of three experiments. *P < 0.05 vs. untreated cells.

The expression of TWEAK and its receptors was assessed under inflammatory conditions in GO (n = 3) and non-GO (n = 3) orbital fibroblast cultures following stimulation with IL-1β or TNF-α by real time-PCR and western blot analyses. Treatment with IL-1β or TNF-α increased *TWEAK*, *TNFR1*, *TNFR2*, and *Fn14* mRNA (Fig 2) levels (P < 0.05) and TWEAK and Fn14 protein levels in a time-dependent manner in both GO and non-GO cells (Fig 3).

### Effect of rTWEAK on pro-inflammatory cytokine production

**Fig 4:**
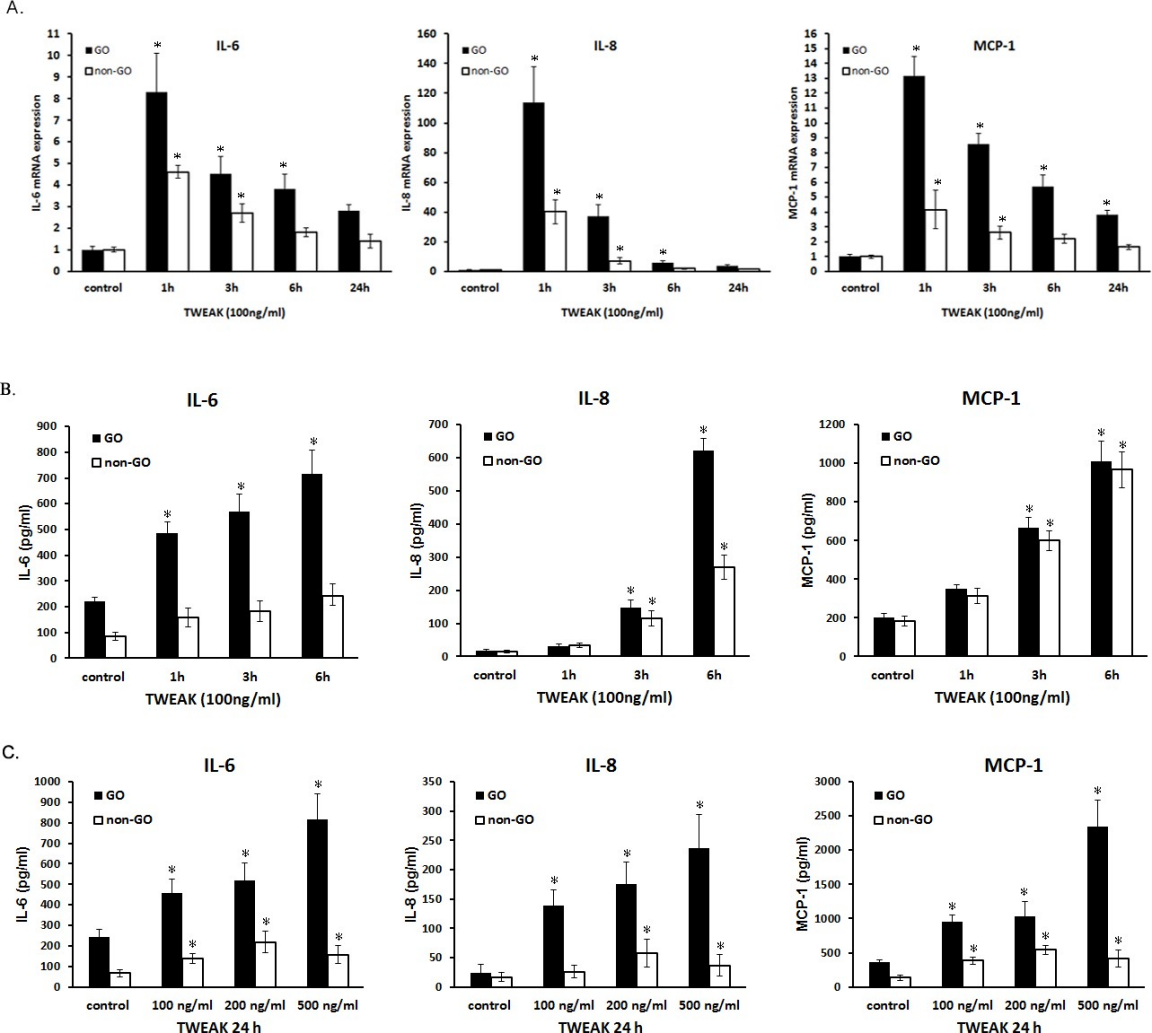
Effect of rTWEAK on mRNA and protein levels of IL-6, IL-8, and MCP-1 in GO and non-GO fibroblasts. GO (n = 3, black columns) and non-GO (n = 3, white columns) orbital fibroblasts were treated with rTWEAK. (A, B) *IL6*, *IL8*, and *MCP1* mRNA levels were quantified by real-time PCR (A), and protein levels were quantified by ELISA (B, C). Data in columns represent mean ± standard deviation. *P < 0.05 vs. untreated control cells.

The expression of pro-inflammatory cytokines including IL-6, IL-8, and MCP-1—which are implicated in the pathogenesis of GO—was examined at the transcript and protein levels by real-time PCR and ELISA, respectively (Fig 4). Treatment with rTWEAK increased *IL6*, *IL8*, and *MCP1* mRNA (Fig 4A) and protein (Fig 4B and 4C) levels in GO cells relative to non-GO cells in a time- and dose-dependent manner.

**Fig 5:**
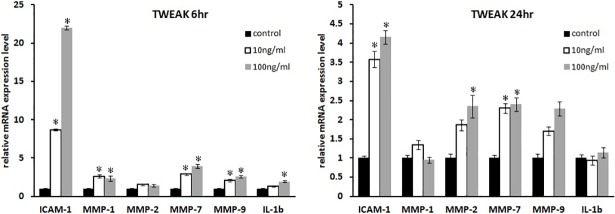
Effect of rTWEAK on *ICAM1*, *MMP1*, *MMP2*, *MMP7*, *MMP9*, and *IL1B* mRNA levels in GO cells. (A, B) GO orbital fibroblasts (n = 3) were cultured and treated with rTWEAK (10 and 100 ng/ml) for 6 h (A) and 24 h (B). *ICAM1*, *MMP1*, *MMP2*, *MMP7*, *MMP7*, *MMP9*, and *IL1B* mRNA levels were evaluated by real-time PCR. Data in columns represent the mean fold difference in relative mRNA level ± standard deviation from triplicate measurements. *P < 0.05 vs. untreated control cells.

The mRNA expression of other cytokines including *ICAM1*, *IL1B*, and MMPs were analyzed in rTWEAK-treated GO cells by real-time PCR (Fig 5). *ICAM1*, *MMP1*, *MMP2*, *MMP7*, and *IL1B* transcripts were upregulated in a dose-dependent manner after 6 h of rTWEAK stimulation (P < 0.05; Fig 5A). In addition, *ICAM1*, *MMP2*, and *MMP7* transcript levels were increased after 24 h of rTWEAK treatment (P < 0.05; Fig 5B).

### Involvement of Fn14 and intracellular signaling pathways in TWEAK-mediated induction of pro-inflammatory cytokine production

**Fig 6:**
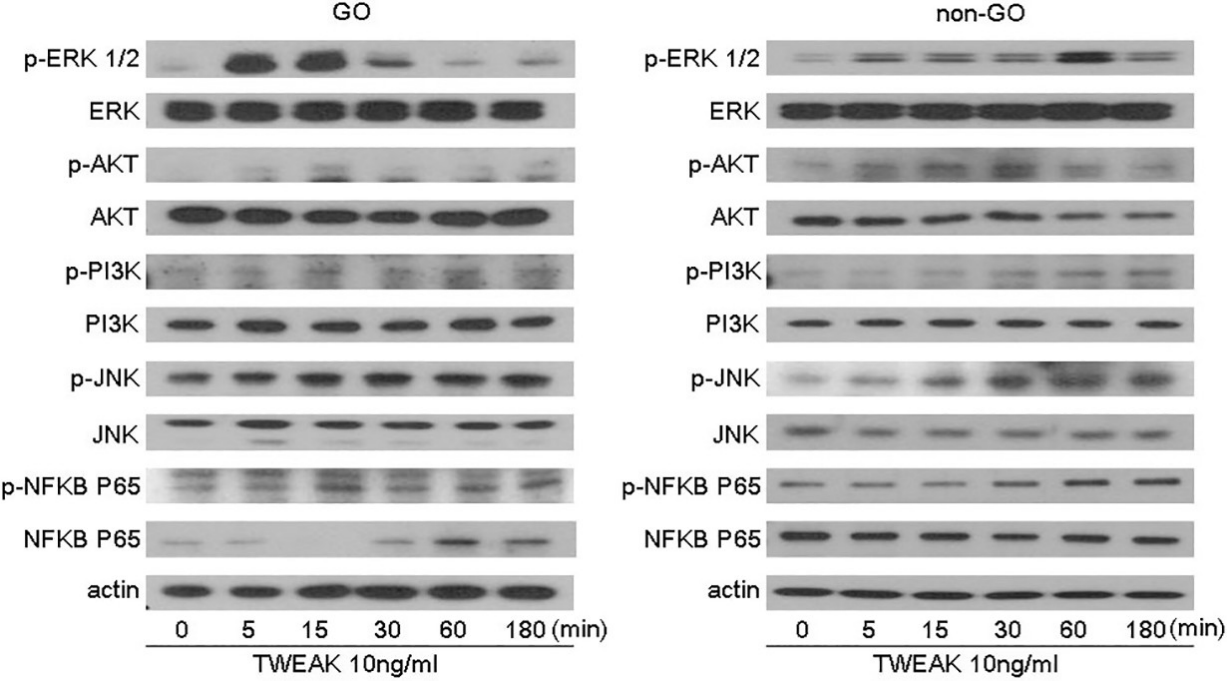
Effect of rTWEAK treatment on intracellular signaling in GO and non-GO cells. Cells were treated with rTWEAK (10 ng/ml) for various times and cell lysates were analyzed by western blotting to assess p-ERK 1/2, p-Akt, p-PI3K, p-JNK, and p-NF-κB p65 levels, with β-actin serving as a loading control.

**Fig 7:**
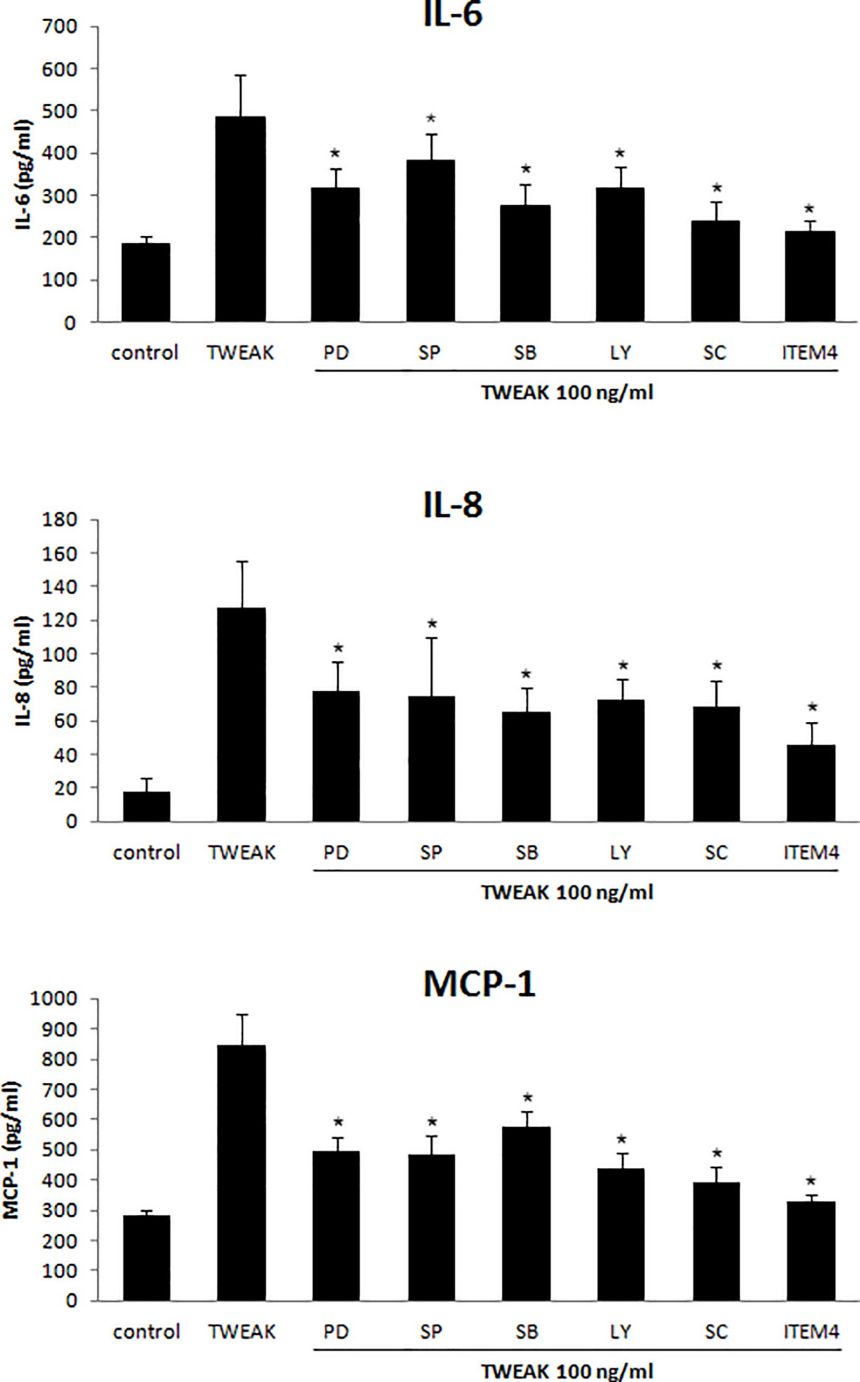
Role of Fn14 and intracellular signaling pathways in rTWEAK-induced pro-inflammatory cytokine expression. IL-6, IL-8, and MCP-1 protein levels in GO orbital fibroblasts (n = 3) pretreated with ERK kinase (PD98059, 20 μM), JNK (SP600125, 20 μM), p38 MAPK (SB203580, 20 μM), PI3K (LY294002, 20 μM), NF-κB p65 (SC514, 10 μM), and Fn14 (ITEM4, 2.5 μg/ml) inhibitor for 1 h followed by rTWEAK treatment (100 ng/ml, 24 h) were measured by ELISA. Data in columns represent mean ± standard deviation of three experiments. *P < 0.05 vs. rTWEAK-treated cells without inhibitor pretreatment.

TWEAK (10 ng/ml) treatment induced the expression of phosphorylated (p-)ERK1/2, p-Akt, p-PI3K, p-JNK, and p-NF-κB p65 within 3 h in both GO and non-GO cells (Fig 6). Pretreatment with 2.5 μg/ml anti-Fn14 mAb (ITEM4) for 1 h or with inhibitors of ERK, JNK, p38 MAPK, PI3K, and NF-κB signaling reduced IL-6, IL-8, and MCP-1 protein expression induced by rTWEAK (100 ng/ml, 24 h) in GO cells (n = 3) (Fig 7). Fn14 blockade most potently suppressed rTWEAK-induced cytokine production. These results indicate that Fn14—a specific TWEAK receptor—and other signaling proteins mediate the inflammatory effect of TWEAK in GO orbital fibroblasts.

### Effect of TWEAK on hyaluronan production

**Fig 8:**
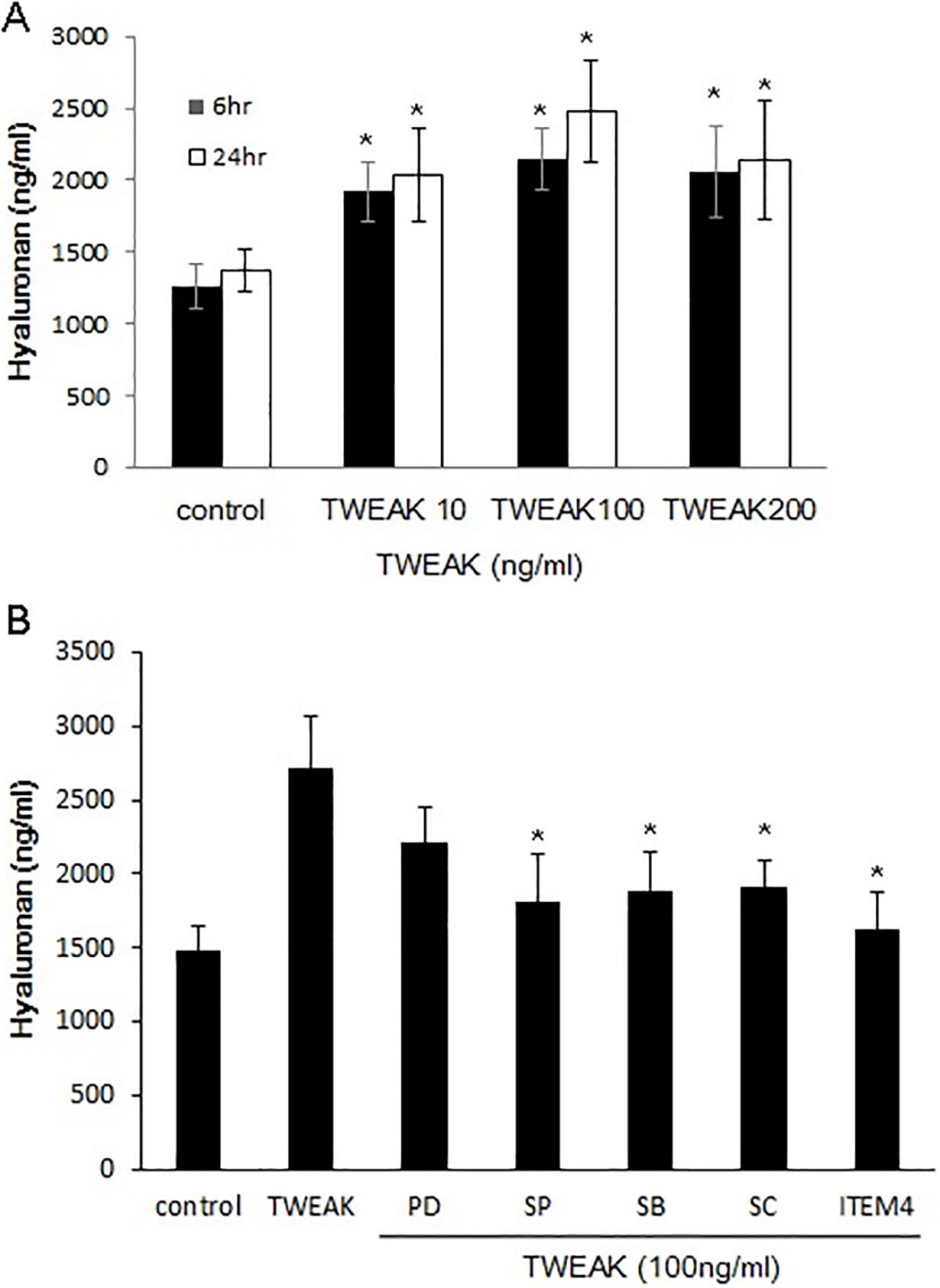
Effect of Fn14 inhibitor on rTWEAK-induced hyaluronan production in GO cells. Hyaluronan (ng/ml) level in GO orbital fibroblasts (n = 3) was measured by ELISA after treatment with rTWEAK (10, 100 and 200 ng/ml) for 6 and 24 h. Cells (n = 3) were pretreated with ERK kinase (PD98059, 20 μM), JNK (SP600125, 20 μM), p38 MAPK (SB203580, 20 μM), NF-κB p65 (SC514, 10 μM), and Fn14 (ITEM4, 2.5 μg/ml) inhibitor for 1 h followed by rTWEAK treatment (100 ng/ml, 24 h). Data in columns represent mean ± standard deviation of three experiments. *P < 0.05 vs. rTWEAK-treated cells without inhibitor pretreatment.

We investigated the effect of TWEAK on the production of hyaluronan, which is an important step in the pathogenesis of GO. The release of hyaluronan into the culture medium following treatment with 10–200 ng/ml TWEAK for 6 and 24 h was measured by ELISA. Hyaluronan level was increased in GO cells upon stimulation with rTWEAK (Fig 8A). Pretreatment with a mAb against Fn14 (ITEM4, 2.5 μg/ml) suppressed rTWEAK (100 ng/ml, 24 h)-induced hyaluronan release (Fig 8B). Inhibition of JNK, p38 MAPK, PI3K, and NF-κB p65 (20 μM, 1 h) pathways similarly reduced hyaluronan production.

### TWEAK protein level is elevated in the serum of GO patients

**Fig 9:**
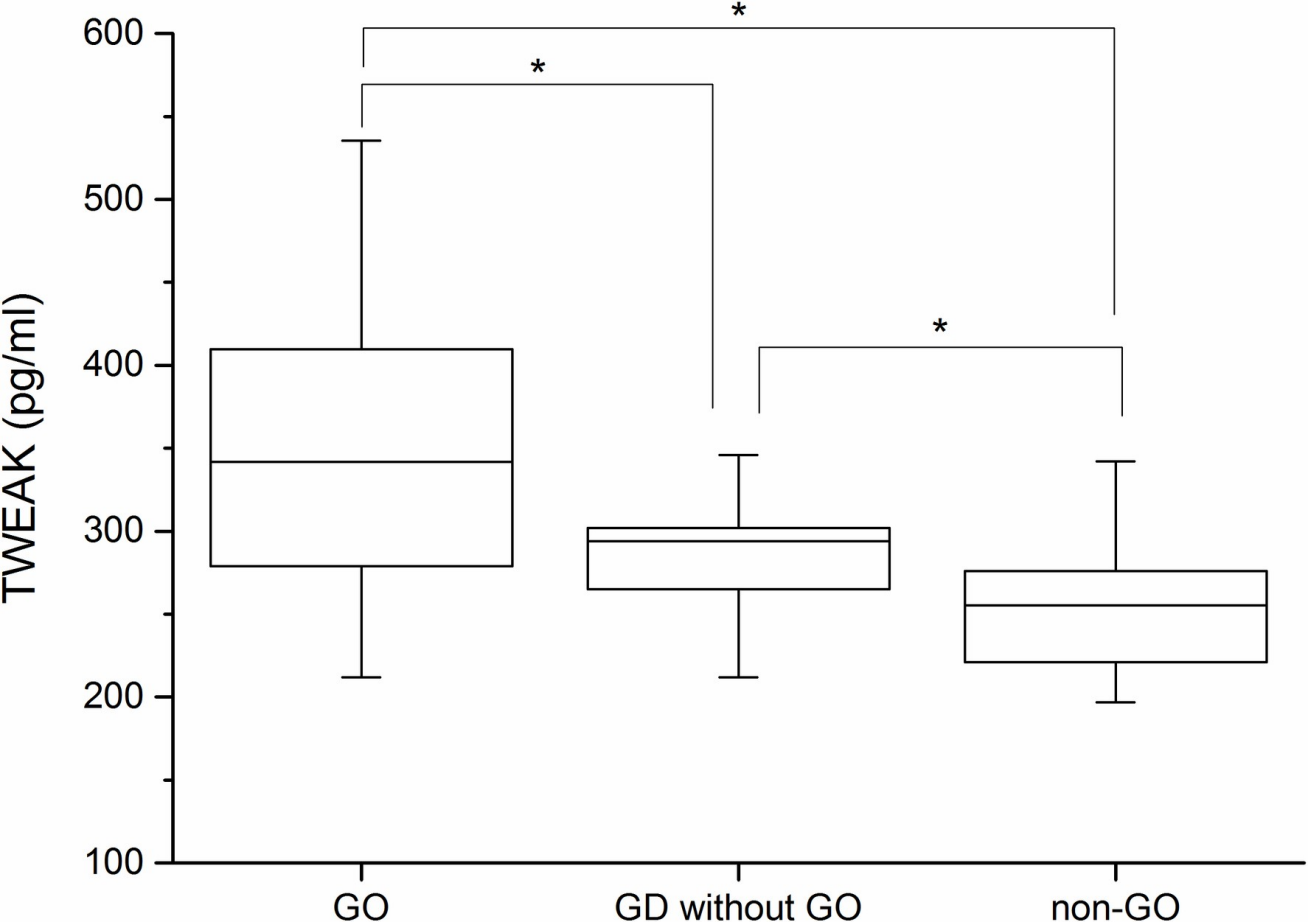
Serum levels of TWEAK in GO patients, GD patients without GO, and normal subjects. Serum levels of TWEAK (pg/ml) were higher in GO patients (n = 56) than in GD patients without GO (n = 35) and healthy subjects (n = 39). Data represent median and interquartile range of values determined by ELISA. *P < 0.05 vs. each group.

We measured serum TWEAK levels in GO patients (n = 56), GD patients without GO (n = 35), and age- and sex-matched healthy control subjects (n = 39). Mean age was similar among groups (GO patients, 36.17 years; GD patients without GO, 34.34 years; and healthy subjects, 32.41 years). Mean serum levels of TWEAK were higher in GO patients (341.86 ± 86.3 pg/ml) than in GD patients without GO (294.09 ± 41.44 pg/ml) (P < 0.05), who had higher levels than healthy subjects (255.33 ± 39.38 pg/ml) (P < 0.05; Fig 9).

We examined the correlation between TWEAK level, CAS, and TSHR Ab level by Pearson’s correlation analysis and found that serum TWEAK level was correlated with CAS (r = 0.629, P < 0.001) and TBII level (r = 0.659 P < 0.001).

## Discussion

The results presented here demonstrate for the first time that TWEAK activates inflammation and hyaluronan production in GO fibroblasts, and that blocking the TWEAK receptor Fn14 abrogates these effects. Cytokine release and hyaluronan production in orbital fibroblasts are important steps in the initiation of exophthalmos and myopathy in the pathogenesis of GO. We showed here that TNF-α and TWEAK levels were higher in GO than in non-GO orbital tissues. Pro-inflammatory cytokines such as IL-1β and TNF-α induced the expression of TWEAK and its receptors TNFR-1, TNFR-2, and Fn14 in GO orbital fibroblasts. Conversely, rTWEAK treatment increased the levels of the pro-inflammatory cytokines IL-6, IL-8, and MCP-1, MMPs, and adhesion molecules such as ICAM-1 in orbital fibroblasts. In addition, hyaluronan production by GO fibroblasts was enhanced by treatment with TWEAK, an effect that was abolished by pretreatment with anti-Fn14 mAb. Fn14—which is induced in multiple cell types during tissue injury—along with some intracellular signaling pathways were involved in TWEAK-induced pro-inflammatory cytokine production. These findings provide evidence for the role of TWEAK/Fn14 signaling in GO pathogenesis.

Serum level of TWEAK was elevated in GO patients as compared to GD patients without GO and non-GO subjects; it was also positively correlated with CAS—a well-known indicator of inflammation—and TBII, which predicts GO activity or severity.[23] Similar TWEAK levels were observed between GO patients with and without radioiodine treatment, (357.33 vs. 337.4 pg/ml; P = 0.484). We presume that the radioiodine did not influence serum TWEAK level since it was administered at least 6 months prior to study enrollment. It was reported that serum TWEAK level was positively correlated with disease activity score-28 in RA patients [19] and was associated with a lower incidence of pulmonary fibrosis and better pulmonary function in systemic sclerosis patients.[24] Although the biological activity of TWEAK is complex, it is known to be activated in inflammatory autoimmune disease and may serve as a reliable diagnostic and prognostic biomarker. Identifying new biomarkers and therapeutic targets other than TSHR for GD and GO by combining microRNA and protein sequencing [25] can improve our understanding of the pathogenesis of these disorders and lead to the development of effective treatments.

Fn14 is a member of the TNFR family [26] that was identified as a functional TWEAK receptor in a cDNA expression library screen [26]. Fn14 is expressed at a low level in various cell types, and is induced under conditions of stress including inflammation. Several growth factors, cytokines, and interleukins are known to induce Fn14 expression in injured endothelial cells, vascular smooth muscle cells, monocytes, and macrophages but not in T and B lymphocytes [26,27]. TWEAK and Fn14 have been implicated in a variety of pathologies and their extracellular localization makes them accessible pharmacological targets, with recombinant soluble variants of or antibodies against these molecules showing high selectivity [24]. It was recently demonstrated using an anti-human Fn14 mAb that TWEAK-induced cell proliferation, migration, and death was mediated by Fn14 [28–30]. TWEAK-specific antibodies that block binding to Fn14 were generated by fusing the ectodomain of Fn14 and the Fc domain of human IgG1. We found that blocking Fn14 using anti-Fn14 mAb suppressed rTWEAK-induced cytokine production and hyaluronan release in cultured GO orbital fibroblasts. Thus, TWEAK/Fn14 signaling plays a protective role in the acute stage of GO-associated inflammation via suppression of cytokine and chemokine release in orbital fibroblasts.

TWEAK activates several signaling pathways that participate in the inflammatory response in injured tissues. NF-κB plays a key role in TWEAK-induced inflammation [31–33]. TWEAK also activates MAPK, ERK, JNK, and p38 pathways [17,34],[35] as well as PI3K/AKT signaling in different cell types. TWEAK induction of JNK is related to activator protein-1 activation [36]. Consistent with these reports, we found that TWEAK induced the phosphorylation of ERK, Akt/PI3K, JNK, and NF-κB p65 in orbital fibroblasts. Furthermore, inhibiting these signaling proteins blocked TWEAK-induced IL-6, IL-8, and MCP-1 expression and hyaluronan production, which was similar to the suppressive effect of Fn14-blocking Ab, indicating that TWEAK/FN14 signaling in the context of GO involves the ERK, Akt/PI3K, JNK, and NF-κB p65 pathways.

## Conclusions

The present study showed that TWEAK is expressed in GO orbital fibroblasts and may contribute to orbital inflammation by inducing pro-inflammatory cytokine secretion. TWEAK level was elevated in the serum of GO patients as compared to GD patients without GO and non-GO subjects, and was positively correlated with clinical inflammation and TSHR Ab titer, indicating that TWEAK is a potential biomarker for diagnosing inflammatory GO. In addition, we showed that inhibiting the TWEAK receptor Fn14 can alleviate TWEAK-mediated GO pathology. Thus, our findings provide evidence that targeting TWEAK/Fn14 signaling may be an effective therapeutic strategy for GO treatment.

## Supporting information

S1 Table Clinical and serological data of patients and controls for serum TWEAK analyses. GD, Graves' disease; GO, Graves' orbitopathy; SD, standard deviation; TSH, thyroid-stimulating hormone; TBII, thyrotropin binding inhibitory immunoglobulin.
(DOCX)

S2 Table List of primers. (DOCX)
