# Supplementary material for: Tumor necrosis factor-like weak inducer of apoptosis induces inflammation in Graves’ orbital fibroblasts
Source: PLoS One. 2018 Dec 21;13(12):e0209583. doi: 10.1371/journal.pone.0209583 (PMC6303076; doi:10.1371/journal.pone.0209583)
Supplement: S1 Table — GD, Graves' disease; GO, Graves' orbitopathy; SD, standard deviation; TSH, thyroid-stimulating hormone; TBII, thyrotropin binding inhibitory immunoglobulin. (DOCX) [file pone.0209583.s001.docx]

Supplementary Table 1. Clinical and Serological Data of Patients and Controls for Serum TWEAK Analyses

|  | GO  (n = 56 ) | GD without GO  (n = 35 ) | Healthy controls  (n = 39 ) |
| --- | --- | --- | --- |
| Gender (Male/Female) | 17/39 | 14/21 | 14/25 |
| Age (years), mean ± SD | 36.17±10.77 | 34.34±11.56 | 32.41±9.88 |
| Smokers, n (%) | 16 (28.6) | 8 (22.8) | 4 (10.2) |
| Duration GD (months), range | 8.31 (1-21) | 9.80 (3-28) | - |
| Radioiodine treatment, n (%) | 12 (21.4) | 2 (5.7) | - |
| Thyroid surgery, n (%) | 6 (10.7) | 2 (5.7) | - |
| T3 (0.58-1.59 ng/dL), mean ± SD | 1.17±0.35 | 1.36±0.27 | 0.73±0.21 |
| free T4 (0.70-1.48 ng/dL), mean ± SD | 1.35±0.33 | 1.20±0.17 | 1.00±0.16 |
| TSH (0.35-4.94 μIU/mL), mean ± SD | 2.01±0.75 | 1.48±1.18 | 2.02±0.35 |
| TBII (0-1.75 IU/L), mean ± SD | 18.31±11.84 | 13.73±10.52 | 0.21±0.35 |

GD, Graves' disease; GO, Graves' orbitopathy; SD, standard deviation; TSH, thyroid-stimulating hormone; TBII, thyrotropin binding inhibitory immunoglobulin
