# Supplementary material for: Tumor necrosis factor-like weak inducer of apoptosis induces inflammation in Graves’ orbital fibroblasts
Source: PLoS One. 2018 Dec 21;13(12):e0209583. doi: 10.1371/journal.pone.0209583 (PMC6303076; doi:10.1371/journal.pone.0209583)
Supplement: S2 Table — (DOCX) [file pone.0209583.s002.docx]

Supplementary Table 2. List of Primers

| Specific primers for SYBR Green PCR reagents (Applied Biosystems, Branchburg, NJ) | |
| --- | --- |
| *Human TWEAK* | 5′-CCC TGC GCT GCC TGG AGG AA-3′ (forward)  5′-AGA CCA GGG CCC CTC AGT GA-3′ (reverse) |
| *Human TNFRSF12A* | 5′-CGC AGG ACG TGC ACT ATG-3′ (forward)  5′-CAG TCC ATG CAC TTG TCC AG-3′ (reverse) |
| human *TNFA* | 5′-GTC TCC TAC CAG ACC AAG-3′ (forward)  5′-CAA AGT AGA CCT GCC CAG ACT-3′ (reverse) |
| human *TNFR1* | 5′-CGG TGG AAG TCC AAG CTC TA-3′ (forward)  5′-TCT AGG CTC TGT GGC TTG TG-3′ (reverse) |
| human *TNFR2* | 5′-GGA AAC TCA AGC CTG CAC TC-3′ (forward)  5′-GGA TGA AGT CGT GTT GGA GA-3′ (reverse) |
| human *TNFR2* | 5′-GGA AAC TCA AGC CTG CAC TC-3′ (forward)  5′-GGA TGA AGT CGT GTT GGA GA-3′ (reverse) |
| *GAPDH* | 5′-ATG GGG AAG GTG AAG GTC G-3′ (forward)  5′-GGG GT CAT TGA TGG CAA CAA TA-3′ (reverse). |
| Catalog numbers of the primers TaqMan universal PCR | |
| *IL6*, | Hs00985639_m1 |
| *IL8* | Hs00174103_m1 |
| *MCP1*, | Hs00234140_m1 |
| *ICAM1* | Hs00164932_m1 |
| *MMP1* | Hs00899658_m1 |
| *MMP2*, | Hs01548727_m1 |
| *MMP7* | Hs01042796_m1 |
| *MMP9* | Hs00234579_m1 |
| *SuIL1B* | Hs01555410_m1 |
| *GAPDH* | H299999905_ml |
